# Rewiring the disordered connectome with circuit-based paired stimulation after stroke—a randomized, double-blind and controlled Phase II trial

**DOI:** 10.1093/braincomms/fcae437

**Published:** 2024-12-04

**Authors:** Xiang-Xin Xing, Jia-Jia Wu, Jiao Qu, Jie Ma, Rong Xu, Yu Zhu, Mou-Xiong Zheng, Xu-Yun Hua, Jian-Guang Xu

**Affiliations:** Rehabilitation Center, Qilu Hospital of Shandong University, Qilu Hospital of Shandong University, Jinan 250012, China; Department of Rehabilitation Medicine, Yueyang Hospital, Shanghai University of Traditional Chinese Medicine, Shanghai 200437, China; Engineering Research Center of Traditional Chinese Medicine Intelligent Rehabilitation, Ministry of Education, Shanghai University of Traditional Chinese Medicine, Shanghai 201203, China; Department of Rehabilitation Medicine, Yueyang Hospital, Shanghai University of Traditional Chinese Medicine, Shanghai 200437, China; Engineering Research Center of Traditional Chinese Medicine Intelligent Rehabilitation, Ministry of Education, Shanghai University of Traditional Chinese Medicine, Shanghai 201203, China; Shanghai Songjiang District Central Hospital, Shanghai Jiao Tong University School of Medicine, Shanghai 201600, China; Department of Rehabilitation Medicine, Yueyang Hospital, Shanghai University of Traditional Chinese Medicine, Shanghai 200437, China; Engineering Research Center of Traditional Chinese Medicine Intelligent Rehabilitation, Ministry of Education, Shanghai University of Traditional Chinese Medicine, Shanghai 201203, China; YangZhi Rehabilitation Hospital, TongJi University, Shanghai 201600, China; Department of Physical Medicine and Rehabilitation, State University of New York Upstate Medical University, Syracuse 13290, USA; Engineering Research Center of Traditional Chinese Medicine Intelligent Rehabilitation, Ministry of Education, Shanghai University of Traditional Chinese Medicine, Shanghai 201203, China; Department of Orthopedics, Shuguang Hospital, Shanghai University of Traditional Chinese Medicine, Shanghai 201203, China; Engineering Research Center of Traditional Chinese Medicine Intelligent Rehabilitation, Ministry of Education, Shanghai University of Traditional Chinese Medicine, Shanghai 201203, China; Department of Orthopedics, Shuguang Hospital, Shanghai University of Traditional Chinese Medicine, Shanghai 201203, China; Engineering Research Center of Traditional Chinese Medicine Intelligent Rehabilitation, Ministry of Education, Shanghai University of Traditional Chinese Medicine, Shanghai 201203, China; School of Rehabilitation Science, Shanghai University of Traditional Chinese Medicine, Shanghai 201203, China

**Keywords:** cortico-cortical paired associative stimulation, stroke, functional connectivity, supplementary motor area, spike-time-dependent plasticity

## Abstract

The cortico-cortical paired associative stimulation, a combined stimulation based on two brain regions, may be an effective strategy for stroke rehabilitation. Our aim was to confirm that the cortico-cortical paired associative stimulation strengthens the connection between brain regions in the motor circuit and promotes improvements in motor function. This was a randomized double-blind, controlled Phase II trial. 44 Stroke patients were treated in a rehabilitation hospital from October 2020 to January 2021 and were randomly assigned to the sham stimulation group and the cortico-cortical paired associative stimulation group. Patients in both groups received 12 days of rehabilitation therapy. Cortico-cortical paired associative stimulation group received one treatment of cortico-cortical paired associative stimulation invention. Both groups received behavioural assessments such as the Fugl–Meyer upper-extremity scale and resting-state functional MRI scans prior to the intervention and on Day 14. 40 patients completed the intervention session. The results of Fugl–Meyer upper-extremity scale showed a more significant improvement in motor function in the cortico-cortical paired associative stimulation group (6.33 ± 1.29) than in the sham stimulation group (3.16 ± 1.38) (*P* < 0.001). The functional connectivity showed that cortico-cortical paired associative stimulation strengthens connections between brain regions. Correlation analysis confirmed that the enhancement of functional connectivity was positively correlated with the recovery of Fugl–Meyer upper-extremity scale (r^2^ = 0.146, *P* = 0.034; r^2^ = 0.211, *P* = 0.0093). The results of functional connectivity suggest that cortico-cortical paired associative stimulation strengthens connections between brain regions. It is expected that this study will provide a positive viewpoint for the neurorehabilitation of stroke patients based on the circuit-level plasticity. (Chinese Clinical Trial Registry: ChiCTR2000036685).

## Teaser

Xing *et al*. reported that they successfully applied the cortico-cortical paired associative stimulation to change the strength of connection between the selected brain areas in post-stroke patients. These outcomes confirmed that the motor function of stroke patients would be recovery by reinforcing the functional connections between the motor-related regions.

## Graphical Abstract

**Graphical Abstract:**
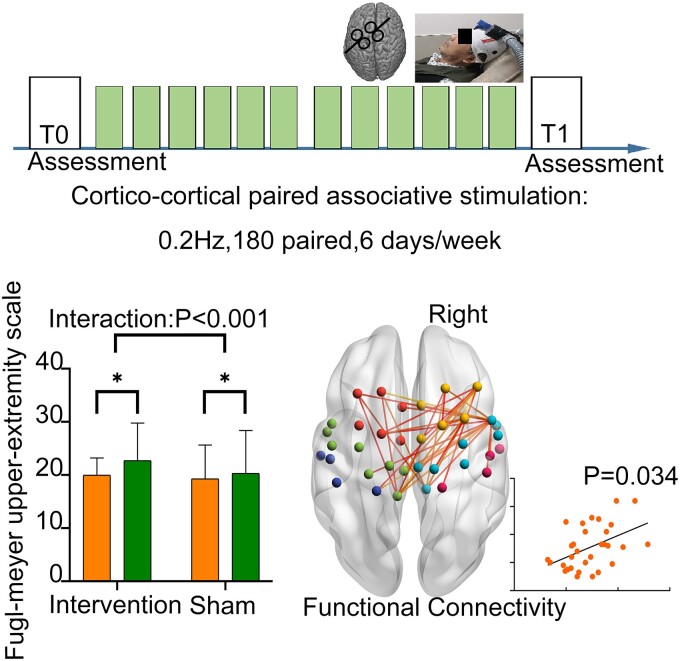

## Introduction

Stroke recovery is a complex process that may involve a combination of spontaneous and learning-dependent processes, including recovery (restoration of functionality to damaged neural tissue), replacement (reorganization of partially spared neural pathways to relearn lost function), and compensation (improvement of the gap between the patient's impaired skills and the demands of the environment).^1^ Although the nature of recovery is varied, it is usually limited by levels of neuronal plasticity and connectivity changes.^2^ These plastic processes are reflected in the remodelling of motor, sensory and language function.^3^ Recently, it has been suggested that the connections determine the functional organization of brain.^4^ Thus, rewiring the disrupted sets of connectome has been considered as a potent strategy for facilitating recovery based on neuroplasticity.

Transcranial magnetic stimulation (TMS) has been shown to induce synaptic plasticity in learning and memory neural circuit to induce plastic alternations similar to Hebbian-like or Spike timing dependent plasticity (STDP) in the intact human brain.^5^ A new modified dual-site TMS based on the STDP mechanism, cortico-cortical paired associative stimulation (ccPAS), is designed to enhance the synaptic efficiency of specific neural circuits in two synaptic efficiencies in connected brain regions.^6^ This is an excellent way to explore changes in neuronal representations and pathways plasticity for specific functions. Therefore, we propose the hypothesis that reconstructing or strength of disrupted neural circuits with ccPAS by selecting appropriate neural pathways can facilitate rehabilitation. The supplementary motor area (SMA) plays an important role in the processes primarily responsible for premotor preparation and voluntary movement.^7^ Anatomical studies have confirmed the presence of dense white matter fibre connections between the SMA and M1^8^ and the stronger coupling between SMA and M1 has been found during the motor task in control normal subjects.^9^ At the same time, coupling decreases after stroke,^10^ and increases with the motor recovery. An experiment with healthy subjects found that paired-pulse stimulation starting from SMA promoted motor evoked potential (MEP) in M1, demonstrating that ccPAS strengthens the connection with the M1 region through activation of the SMA.^11^ These studies provide an anatomical and theoretical basis for the design of the present study.

In this study, we investigated whether ccPAS could improve the motor recovery and whether it could re-establish connections in the brain after stroke. We hypothesized that the connection between two brain regions in the motor circuit could be strengthened by stimulating a pair of cortical regions with fixed timing lag, and that the strengthening of connection could promote the recovery of motor function. Functional connectivity (FC) is a metric of FC that assesses the strength of connections. We also performed a correlation analysis between FC and motor function after intervention.

## Materials and methods

### Participants

Stroke patients were collected in YangZhi Rehabilitation Hospital between October 2020 and January 2021. Inclusion criteria for all participants: (i) patients were diagnosed as stroke by clinical assessment (Chinese Guidelines for the Diagnosis and Treatment of Acute Ischemic Stroke 2018 and The Diagnostic criteria of Integrated Traditional Chinese and Western Medicine for Cerebral Infarction and Cerebral Haemorrhage)^12^; (ii) age ≥18 and ≤70, with no gender restriction; (iii) duration of disease ≥3 months and ≤12 months; (iv) MRI or CT showing haemorrhagic cerebral infarction or ischaemic stroke. The lesion is located in unilateral cerebral hemisphere, and the lesion is not located in the M1 and SMA; (v) primary school education or above, with normal cognitive function (Mini-mental state examination corresponding to critical value above different education level). Exclusion criteria: (i) severe heart, liver and kidney diseases or infectious diseases; (ii) history of epilepsy, psychiatric disorders, or other severe cognitive or emotional dysfunction diseases; (iii) patient has MRI and TMS prohibited implants in the body; (iv) recent use of prophylactic or antiepileptic and cognitive improvement drugs; (v) pregnant or lactating women.^13^

The study was approved by the Ethics Committee and the patients or their families have signed an informed consent from (Chinese Clinical Trial Registry: ChiCTR2000036685).

### General procedure

#### Randomization, blinding and sample size

The patients were randomly divided into two groups in a ratio 1:1 by the method of random number table. Each participant was given a particular number in lieu of replace the real name. The allocation sequence was kept confidential from the therapists, evaluators and the participants and their caregivers. The allocation sequences were placed in opaque sealed envelopes with restricted access, which were given the day before to the researcher responsible for implementing the intervention. The participants were divided into a ccPAS stimulation group (ccPAS group) and a sham stimulation group (sham group). The blinding process was done independently by executive. If blinding failed, the subject was removed from the study protocol. The evaluator must identify particular participant by number. We arranged for data analysis by an independent researcher who was not involved in recruitment, screening, evaluation or intervention. The sample size was calculated based on the results of our preliminary experiment with Fugl–Meyer upper-extremity scale (FMA-ue), with a required sample size was 17 participants in each group. If a 20% dropout rate is taken into account to compensate for subsequent losses, a minimum of 42 participants would be required to reach the recruitment target of 21 per group.^12^

#### Interventions

Each participant received routine rehabilitation and multi-disciplinary medications for 2 weeks, 6 days per week. Routine rehabilitation services mainly consisted of daily physical therapy and occupational therapy by the same experienced therapist. Specific treatment was tailored to each patient's level of function and individual needs.

#### Evaluation of motor cortex excitability

For many stroke patients, especially those with serve motor deficits, obtaining reliable resting-motor threshold (RMT) from the lesioned hemisphere may be challenging or infeasible. This difficulty arises because the lesioned hemisphere may not produce consistent or detectable evoked MEP at standard stimulation intensities. Therefore, using RMT from the contralateral hemisphere provides a more feasible and reliable method for setting a baseline for TMS intensity. Using 80% of RMT of the contralateral hemisphere helps to ensure that the stimulus intensity is within a safe and effective therapeutic range. This intensity is typically sufficient to induce neuromodulation in the lesioned hemisphere without risking overstimulation. This approach is supported by previous research in the field of neuromodulation and stroke rehabilitation. Studies have shown that TMS targeting the lesioned hemisphere using intensity parameters obtained from the contralateral hemisphere is effective in facilitating motor recovery.^14^

Before interventions, the excitability of M1 in the contralateral hemisphere was assessed with MEP by monopulse TMS. The equipment was a MagTD magnetic field stimulator from Wuhan YIRUIDE Group Co., LTD. (Wuhan, China). All the assessments were completed by the same physician, and the timing of assessment was essentially the same across subjects. Participants were asked to remain awake during the assessments.

For the motor representative region of the first dorsolateral interosseous muscle in the healthy hand, the identified MEPs were continuously extracted 5 out of 10 times. MEPs were recorded using surface electrodes positioned in a belly tendon montage. RMT was defined as the minimum output intensity, eliciting at least 5 out of 10 MEPs higher than 100 µV.^11^

#### ccPAS group

During the ccPAS approach, subjects were asked to lie comfortably in a recliner with both forearms resting on pillow that had been placed on their legs beforehand. The head was rested against the back of the chair for support in a completely relaxed posture.

##### Stimulus sites

Before intervention, subjects wore the localization cap of a standard 10–20 EEG system (Wuhan YIRUIDE Group Co., LTD.) to determine the location of SMA and M1. An 8-shaped coil was placed at the M1_hand_ in the lesioned hemisphere, symmetrical to the site where MEP was evoked when the stimulus intensity was measured in the healthy hemisphere. The coil was tangent to the scalp, and the midline was rotated 45° so that the induced current in M1 was conducted from back to middle to forward, which was considered as the most therapeutically effective location. Another 8-shaped coil was placed on the SMA of the lesioned hemisphere, with the position of the SMA placed 3 cm in front of the Cz and marked with a waterproof marker.^11^

##### Intensity, frequency, number of stimulation and others

According to Arai *et al.*, the stimulation intensity was set as 80% RMT for M1, 120% RMT for SMA and the stimulation interval from SMA to M1 was set as +6 ms.^11^ The once-daily ccPAS stimulation consisted of 180 paired TMS pulses at a frequency of 0.2 Hz, and each session stimulation lasted for 15 min.^15^

##### Sham stimulation group

Two coils were placed vertically on the participant's scalp during sham stimulation. The coils produced sound without effective stimulation of the cerebral cortex.

### fMRI scans and pre-processing

All MRI scans were performed in the MRI room of Songjiang District Central Hospital of Shanghai. The MRI system is a 3.0T tandem uMR 770 MR scanning Imaging system (United Imaging, Shanghai, China) with a standard cranial 32-channel coil. To avoid differences in resting brain activity at different times of the day, the MRI scanning time all subjects were restricted to 16:30 and 19:30 p.m. During the scanning procedure, subjects were placed in a supine position with eyes closed, earplugs to minimize noise interference and head fixed in the coil. Patients were asked to remain awake, breathe calmly and try not to move. fMRI data were collected at baseline and 2 weeks later.

All patients underwent resting fMRI (EPI_BOLD) covering the whole brain. Resting-state fMRI were acquired using a planar echo sequence (EPI_BOLD). The scanning parameters were as follows: slice number = 43; matrix size = 64 × 64; FOV = 240 × 240 mm^2^; TR/TE = 3000/30 ms; flip angle = 90°; slice thickness = 3.0 mm; voxel size = 3.2 × 3.2 × 3.40 mm^3^; number of repetitions = 240 for a total acquisition time of 12 min.

Data pre-processing procedures were performed using the Statistical Parametric Mapping 12 (SPM 12) toolbox (http://www.fil.ion.ucl.ac.uk/spm/) based on the MATLAB 2013b platform (The Mathworks, Inc, Natick, The Statistical parameters of US). To eliminate unstable signals, we removed the first 10 volumes. For participants with an affected left hemisphere, the brain image were flipped from left to right in order to localize the affected side of the brain to the right hemisphere side.^16^ Subsequent pre-processing steps included slice timing, head motion correction, coregistration with individual anatomical images, spatial normalization with the Montreal Neurological Institute spatial EPI template, resampling to 3.0 × 3.0 × 3.0 mm^3^ and smoothing using a 6-mm full-width at half-maximum Gaussian kernel. Linear detrending and bandpass filtering (0.01–0.08 Hz) were then performed. Finally, regressions were performed on the nuisance signals in the data (including the averaged signal from white matter, cerebrospinal fluid and Friston 24 head motion parameters). Images with excessive head motion (more than 2° or 2 mm) or serious artefacts were discarded.

#### Functional connectivity and extracted ROIs

Since we mainly focused on the alternations in motor function of the patients, the motor-related cortex were selected, including the S1, M1, premotor cortex and SMA. The Human Brainnetome Atlas is a brain atlas subdivided on the basis of the Brodmann atlas, with a total 246 regions.^17^ These include A1/2/3ulhf (upper limb, head and face region), A1/2/3Tonia (tongue and larynx region), A2 (brodmann area 2), A1/2/3TRU (trunk region) of primary sensory cortex, the A4hf (head and face region), A6cdl (caudal dorsolateral area 6), A4ul (upper limb region), A4t (trunk region), A4tl (tongue and larynx region), A6cvl (caudal ventrolateral area 6), A1/2/3LL (lower limb region), A4ll (lower limb region) of M1, the A8 m (medial area 8), A8dl (A8dl, dorsolateral area 8), A6dl (dorsolateral area 6), A6 m (medial area 6), A8vl (ventrolateral area 8) and A6vl (ventrolateral area 6) of SMA and pre-motor area. A total of 36 sub-regions were used as regions of interest (ROIs) and binary masks were produced accordingly. The mean time series of each ROI was extracted by averaging the time series of BOLD signal and then correlated to the time series of other voxels across the brain in terms of ROIs using the pre-processed functional images. The resultant correlation maps were subsequently normalized using the Fisher’s r-to-z transform.

### The outcome

The total score of the FMA-ue from baseline to the end of 2 weeks was the primary outcome.^18^ The scale is widely used to evaluate upper extremity motor function with a high degree of reliability and popularity. It has a total of 66 points divided into 33 items with a maximum of 2 points and a minimum of 0 points for each item. The higher the scores, the better the motor performance. Assessment of the clinical scale is performed by the same senior physician. The FC was the secondary outcome.

### Safety considerations and adverse effect

Possible adverse events associated with the study included seizures, skin abrasions, muscle strain, muscle acid, hypoglycaemia, dizziness, headache, nausea and localized skin redness and itching. Any accidental injuries and sudden diseases that occurred during the study were recorded in detail as adverse events.

### Statistical analysis

For the continuous variable obtained, normality test was performed first. Normally distributed continuous variable were described as mean ± standard deviation (X ± S). Paired *t*-test was performed to compare pre- and post-intervention data within the same group. Comparisons between groups involving pre- and post-intervention data were performed by 2-way repeated measures ANOVA (2-way repeated measures ANOVA). There were two levels of inter-group factors, the stimulus group and the sham stimulus group. The intra-group factor was the time of assessment, also at two levels, before and after treatment. The main effect and inter-action between the two groups were analysed. For measurement data of non-normal distribution, Wilcoxon signed-rank test was used for comparison between the two groups. Statistical significance level was set as α = 0.05. Inter-group sex and affected side were compared by χ^2^. *P* < 0.05 was considered statistically significant. SPSS22.0 software was used for statistical analysis (IBM, Chicago, US). And then, the Pearson’s correlation analysis was performed between post-intervention FC value and the post-intervention FMA-ue of the ccPAS group. Meanwhile, using the minimal clinically important difference (MCID) as an indicator, clinicians and researchers can determine whether the change in outcome scores after the intervention indicate meaningful and clinically important improvement for patients. Previous studies have shown that a score of 4.6 can achieve the MCID.^19^

## Results

### General information and clinical data

**Figure 1:**
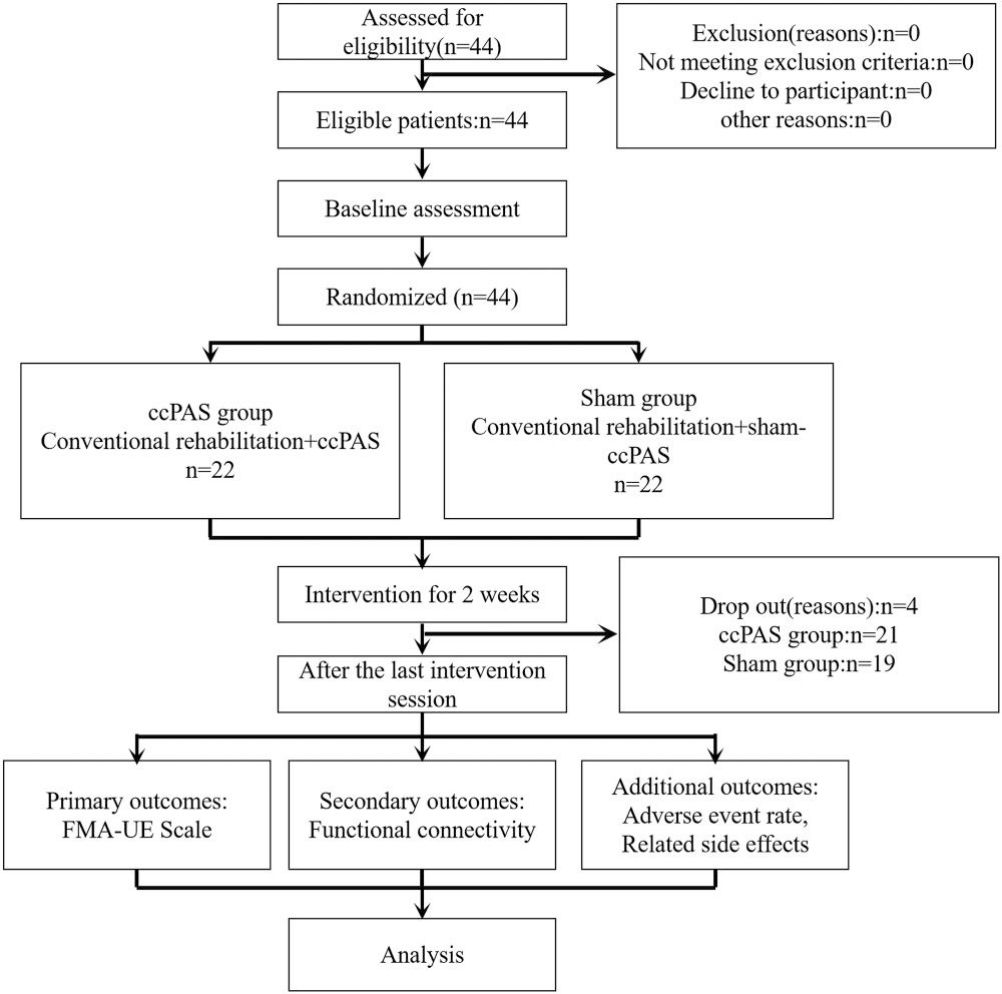
The clinical flow chart of this study.

A total of 44 participants were enrolled for the study, 4 patients withdrew after enrolment (1 in the ccPAS group and 3 in the sham group), and 40 patients completed intervention course. There were 21 cases in ccPAS group and 19 cases in sham group. No significant difference was found in clinical characteristics of the patients in the two groups before interventions (all *P* > 0.05) (Fig. 1; Table 1).

**Table 1:** General information and clinical data of enrolled participants

	ccPAS group (*n* = 21)	Sham group (*n* = 19)	*P*
Age (years)	50.67 ± 12.76	49.84 ± 10.69	0.827
Gender (male/female)	19/2	17/2	1.000
Course of disease (month)	7.10 ± 3.35	7.16 ± 3.67	0.955
Stroke type (infarction/haemorrhage)	13/8	15/4	0.407
Side of lesion (left/right)	8/13	12/7	0.205
MMSE	27.29 ± 2.81	27.26 ± 2.89	0.980
RMT	44.5 ± 10.3	48.1 ± 7.0	0.212

ccPAS, cortico-cortical paired associative stimulation; MMSE, mini-mental state examination; RMT, resting-motor threshold.

### Clinical assessment

**Figure 2:**
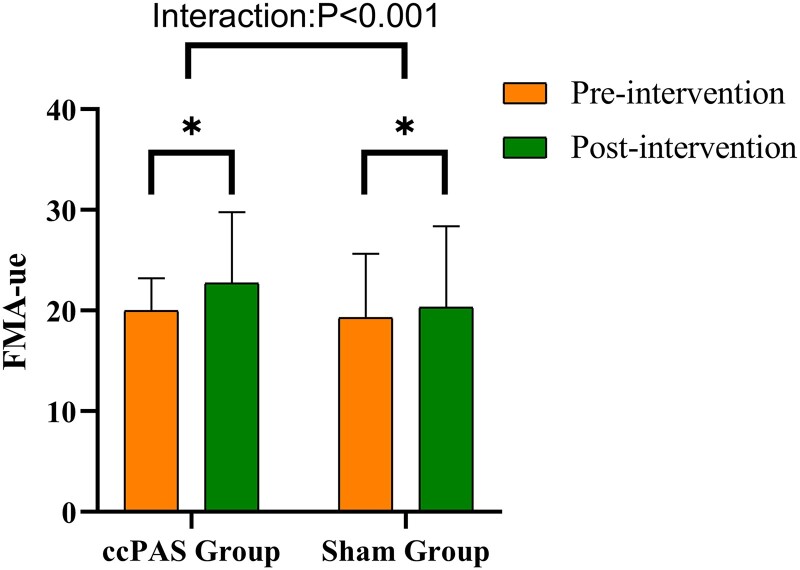
**Comparison of FMA-ue before and after treatment between the two groups.** There were significant differences between group times (*P* < 0.001 and F = 596.23). Pre- and post-treatment comparisons within the group were significantly different (*P* < 0.05, tccPAS = 22.71 and tsham = 9.93).

There was no significant difference in FMA-ue score between the two groups before treatment (*P* > 0.05). Repeated measures ANOVA at the end of treatment showed a significant increase in FMA-ue scores in both groups compared with those before treatment (time main factor, *P* < 0.001). The interaction effect of group × time was significant (time main factor, *P* < 0.001). *Post hoc* tests showed a significant difference in the increment of FMA-ue between the ccPAS group (6.33 ± 1.29) and sham group (3.16 ± 1.38) (*P* < 0.001) (Fig. 2; Table 2). And the FMA-ue scores in the ccPAS group improved 6.33 (95% CI: 5.04 to 7.62) compared with the sham group 3.21 (95% CI: 1.98 to 4.44). Our study showed that 90% of patients in the ccPAS group could achieve MCID in the ccPAS group, whereas only 16% of patients in the sham group could achieve MCID.

**Table 2:** Changes in FMA scores before and after 2 weeks of clinical treatment

Group	Time point	FMA-ue
ccPAS group（*n* = 21）	Pre-intervention	22.57 ± 16.33
	Post-intervention	30.47 ± 16.63*#
Sham group（*n* = 19）	Pre-intervention	25.79 ± 13.10
	Post-intervention	28.95 ± 12.96*

ccPAS, cortico-cortical paired associative stimulation; FMA-ue, Fugl–Meyer upper-extremity scale.

*P < 0.05 for pre- and post-treatment comparisons within groups, #*P* < 0.05 for incremental comparisons between ccPAS group and sham groups in post-treatment comparisons for post-hoc test.

### Functional connectivity

**Figure 3:**
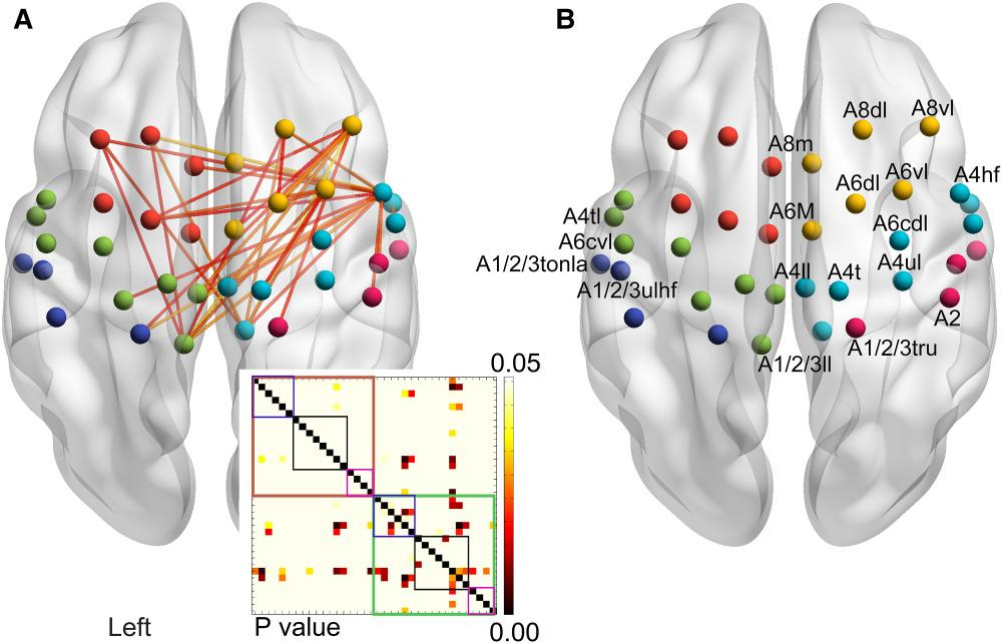
**Analysis of FC between the two groups after treatment.** The (**A**) is the stereogram of the *t* value map, yellow ball means the right SMA/PMC sub-region, the cyan ball means the right M1 sub-region, the *P* ball means the right S1 sub-region, the red ball means the left SMA/PMC sub-region, the green ball means the left M1 sub-region, the navy blue ball means the left M1 and the connections between balls mean the strength of FC. Inside the contralateral hemisphere, the FCs between the SMA/PMC and M1 were increased (A8dl-A1/2/3LL *P* = 0.038, A8vl-A1/2/3LL *P* = 0.044). Inside the affected side, the FCs were increased in the SMA/PMC (A6dl-A8dl *P* = 0.047, A6dl-A8vl *P* = 0.008, A6dl-A6vl *P* = 0.022, A6m-A8vl *P* = 0.049), M1 (A4hf-A4tl *P* = 0.013, A6cvl-A1/2/3LL *P* = 0.031, A6cvl-A4ll *P* = 0.024), SMA/PMC between the M1 (A8m-A6cvl *P* = 0.019, A8dl-A6cvl *P* = 0.013, A8dl-A1/2/3LL *P* = 0.012, A8vl-A1/2/3LL *P* = 0.002, A8vl-A4ll *P* = 0.013, A6vl-A1/2/3LL *P* = 0.020, A4t-A6vl *P* = 0.047), SMA/PMC between S1 (A8vl-A1/2/3TRU *P* = 0.034) and M1 and S1 (A6cvl-A1/2/3ulhf *P* = 0.019, A6cvl-A2 *P* = 0.028). Between the hemispheres, the FCs were increased between the affected SMA/PMC and contralateral M1 (A8dl-A6cvl *P* = 0.027, A8dl-A6cvl *P* = 0.004, A8dl-A1/2/3LL *P* = 0.011, A8vl-A4ll *P* = 0.028, A8vl-A6cvl *P* = 0.035, A6dl-A6cvl *P* = 0.048), SMA/PMC (A6dl-A8vl *P* = 0.038, A6vl-A6vl *P* = 0.018), results also occurred between the M1 (A1/2/3LL-A8dl *P* = 0.048, A4ll-A8vl *P* = 0.013, A1/2/3LL-A8vl *P* = 0.004, A1/2/3LL-A6vl *P* = 0.019, A1/2/3LL-A6cvl *P* = 0.009, A4ll-A6cvl *P* = 0.014, A6cvl-A4ul *P* = 0.036) and S1 (A1/2/3TRU-A8vl *P* = 0.035, A1/2/3TRU-A6cvl *P* = 0.006) regions on the affected hemisphere and those on the contralateral hemisphere. There were no significant differences observed in FC within the S1 (*P* > 0.05). The image in the lower right corner is a *P* value map, the *X* and *Y* axis means the 36 brain regions. The Brown box means the left hemisphere, the green box means the right hemisphere, the blue box means the SMA and the premotor cortex, the black box means the M1, and the purple box means the S1. The (**B**) shows each sub-region.

The results showed significant differences in the main and interaction effects of FC within the ROIs (*P* < 0.05). There was an overall significant increase in FC in the post-intervention ccPAS group compared to the post-intervention sham group. In the contralateral hemisphere, FC increased between the SMA/PMC and M1 (*P* < 0.05). In the lesioned side, FC increased in the SMA/PMC, M1, SMA/PMC between the M1, SMA/PMC between S1 and M1 and S1 (*P* < 0.05). Between the hemispheres, there was an increase in FC between the affected SMA/PMC and contralateral M1, SMA/PMC (*P* < 0.05). The same results were observed between the M1 and S1 regions of the affected hemisphere and those on the contralateral hemisphere (*P* < 0.05). There were no significant differences observed in FC within the S1 (*P* > 0.05) (Fig. 3).

### Correlation between clinical functional assessment and functional connectivity

**Figure 4:**
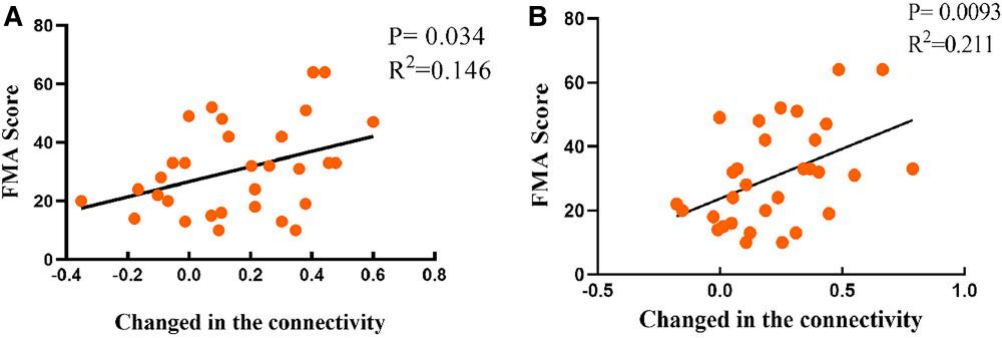
The correlation between the FMA-ue score of FC between the A6cvl and A8dl (A) and the A6cvl and A8vl (B).

Correlation analysis showed that FC between A6cvl and A8dl (r^2^ = 0.146, *P* = 0.034) and A8vl (r^2^ = 0.211, *P* = 0.0093) in affected hemisphere were positively correlated with post-intervention FMA-ue score (Fig. 4).

### Adverse events

Four participants withdrew from the experiment. Among them, two participants reported scalp pain during TMS assessment, and one participant was unable to tolerate the noise of fMRI, and another participant withdrew from the experiment without any reasons. During the intervention, one patient reported dizziness and discomfort, which may have been due to change in the patient’s prescription for treatment of hypertension during hospitalization. After stabilization of blood pressure, the intervention continued without significant discomfort. Intervention resumed without apparent discomfort after the blood pressure stabilized. The remaining participants did not report obvious discomfort (Table 3).

**Table 3:** Overall summary of adverse events

	Statistics	ccPAS (*N* = 22)	sham ccPAS (*N* = 22)
Seizures	*N* (%)	0 (0%)	0 (0%)
Skin abrasions	*N* (%)	0 (0%)	0 (0%)
Muscle strains	*N* (%)	0 (0%)	0 (0%)
Muscle acid	*N* (%)	0 (0%)	0 (0%)
Hypoglycaemia	*N* (%)	0 (0%)	0 (0%)
Scalp pain	*N* (%)	0 (0%)	2(9.1%)
Dizziness	*N* (%)	1 (4.5%)	0 (0%)
Headache	*N* (%)	0( 0%)	0 (0%)
Nausea	*N* (%)	0 (0%)	0 (0%)
Localized skin redness and itching	*N* (%)	0 (0%)	0 (0%)

## Discussion

Different from previous rTMS intervention protocol for single brain regions, we successfully applied the new protocol to reconnect the disorganized connectome between the brain regions in post-stroke patients. This is closely related to the physiological connection between the SMA and M1 as well as the long-term potentiation (LTP) mechanism induced at specific time periods. The results of increased FC revealed that were by ccPAS intervention enhanced brain connectome. Meanwhile, it was proportional to the increased motor function scores in stroke patients. These outcomes confirm that motor function in stroke patients by strengthening the functional connections between the motor-related regions. Additionally, the ccPAS may be the beneficial and valuable TMS protocol with positive and effective therapeutic effects on stroke rehabilitation.

Overall activity in the cerebral cortex is significantly reduced in stroke patients compared with intact undamaged brain.^20^ Function is a property of the inter-actions between brain regions, and there is a growing consensus in the field of neuroscience on this point.^21^ Previous study had confirmed that behavioural deficits in stroke patients are not only due to local tissue damage, but also due to altered connectome among structurally intact regions that are connected to the damaged site.^22^ Several investigators have conceptualized the recovery process as one aimed at increasing the efficiency of circuit utilization.^23^ It is related to the synaptic plasticity, which is central to the recovery progress after stroke. Synaptic plasticity refers to the long-term alternation of neuronal connection strength with experience, the ability to mediate synaptic remodelling during development, learning, and memory, and the ability to respond to the external stimulus, internal medium, injuries and other neurological disorders.^24,25^ Donald Hebb has articulated that strength of inter-cellular connections is enhanced if the cell A firing the cell B repeatedly.^26^ This is a powerful mechanism for shaping and modifying the response characteristics of neurons.^25^ Subsequent studies have found this input–output plasticity is mediated by the *N*-methyl-D-aspartate glutamate receptors (NMDARs), which require a coincidence of glutamate binding and membrane potential depolarization for gating.^27^ At the level of cellular machinery, studies have showed that signalling pathways of STDP are modulated by the NMDA receptor-dependent LTP, NMDAR-dependent long-term depression (LTD) and the Metabotropic glutamate receptor-dependent and/or cannabinoid Type 1 receptor-dependent LTD.^28^ The three mediated mechanisms provided critical components of plasticity for post-synaptic depolarization. Most synaptic plasticity forms investigated to date fall into the standard autonomous correlative Hebbian category, which includes practically all types of STDP, which are the quintessential Hebbian protocol for experimentally induced LTP or LTD.^29,30^ Indeed, STDP retains the fundamental autonomous correlation-based property, while adding temporal requirements that somewhat refine and extend its capabilities.^31,32^ STDP is bidirectional and sequential, with pre-before-post spikes induces LTP, and post-before-pre spikes induces LTD. It is induced by the precise temporal windows.^33^ Over the past few decades, extensive researches have been conducted in various animal models to explore the learning rule. Standard pairwise monitoring of the excitatory post-synaptic potential (EPSPs) in layer 2/3 pyramidal neurons of rat visual cortical slice has shown that the dependence of synaptic modification is caused by the interval between the pre- and post-synaptic spikes.^34^ The LTP effect could be observed after 60–80 pairings from the pre- to post-synaptic spikes.^35^ It selects input pathways based on the spike-time correlations and support the formation of neuronal FC.^36^ In a word, it changes the strength of the connection/coupling between the two brain regions. In-depth studies have found that the STDP-based neural architectures have joint feed-forward excitatory and inhibitory function, even in human brain structures.^37^

Markram *et al*. found that LTP is induced when EPSPs were followed by post-synaptic action potentials within 100 ms.^29^ By testing the significant temporal effects between EPSPs and post-synaptic action potentials, the investigators explained the apparent relationship between the magnitude of synaptic enhancement and the time of stimulus intervals,^30^ and they revealed that enhanced synaptic connection can be induced in different couple of brain regions, both *in vivo* or *in vitro*.^24^ Thus, STDP is considered as contributing to the formation of neural circuit.^38^ One mathematical model also suggests that the dynamic characteristics of the neuron system could be significantly changed by adjusting the time interval, which results in different connection patterns and changes the synchronization characteristics between inter-connected neurons.^39^ In the field of stroke rehabilitation, increasing attention has been paid on facilitating the recovery of brain dysfunction, and it is believed that the reorganization of the cortices contributes to the motor rehabilitation of the paralysed limb. Meaningfully, this neuro-modulatory mechanism possibly plays an important role in the reconstruction process.

TMS is one of neuromodulation methods that is believed to function in the reconstruction of damaged pathways.^6^ According to Vlachos’s experiments, stimulated synapses show coordinated excitatory in terms of both structural and functional plasticity.^40^ They provided the evidence that synaptic strength is excitatory after rTMS, and the excitability is prolonged. Meanwhile, study of the voxel-based morphometry showed that the grey matter in brain regions thinned after several sessions of rTMS. It demonstrated that the TMS could cause the dynamic structural alternations in cortical plasticity and powerful changes in synaptic activity.^41^ Whereas, rTMS is mostly single-target therapy which always causes connections changes between the different brain regions.^23^ As the study of TMS deepened, Stefan *et al*. paired somatosensory electrical stimuli with TMS stimuli at specific stimulus intervals and found that the motor cortex was significant activated.^42^ This experiment provided a direct link to the basic physiology of cortical neurons, called paired associative stimulation (PAS). Based on the protocol of PAS, ccPAS applied repetitive paired stimuli to two different cortical regions at a few millisecond interval to study the physiological inter-actions between the brain regions. Koganemaru *et al*. co-stimulated homologous regions in the left and right M1 region induced to a long-term increase in MEP in M1 region of condition stimulation.^43^ Subsequently, ccPAS protocol was developed to study STDP phenomena within the inter-hemispheric cortical network, which induces purposeful bidirectional modulation of cortical plasticity in the target region.^11^ To date, ccPAS in bilateral M1,^44^ ventral pre-motor area and M1, SMA and M1, and posterior parietal lobe and M1 have been proved to be effective in inducing STDP, LTP and LTD like effects.^11^ Therefore, ccPAS is thought to be used to drive reorganization of specific cortico-cortical pathways, thereby promoting functional recovery.^45^

Based on previous studies, we used the reasonable stimulus protocol described above to intervene in stroke patients. The results, as assessed by FC and clinical function, confirmed our hypothesis that the intervention of ccPAS targeting M1 and SMA could significantly improve the upper limb motor function in stroke patients. While, different to the previous investigators, we pay more attention on the strength of brain connection than on changed activity of the brain region receiving condition stimulation. Moreover, the results of correlation analysis revealed that enhancement of connection between specific motor-related brain regions could improve the recovery of motor function. Therefore, we made further assumption that the clinical assessment of behaviour could be effectively influenced by changing the segmental strength of relevant functional neural circuits. This warrants exploration in more neural circuit or segments.

Limitations of this study include the fact that we only used a stimulation frequency of 0.2 Hz to investigate the impact of enhanced connectivity on central plasticity and motor function recovery in stroke patients. To offer a more comprehensive perspective on therapeutic effectiveness, we intend to broaden our evaluation scope in future experiments. Additionally, exploring the potential benefits of high-frequency ccPAS in promoting the motor function recovery of stroke is a worthy direction for long-term in-depth research. We plan to conduct experiments using relevant animal models to deepen understanding.

Our findings could play an important role in promoting the motor recovery after stroke. After a specific time interval of ccPAS stimulation, the connectivity between the motion-related brain regions were remodelled and strengthened. It further confirms that ccPAS improves motor function by effectively reconstructing or enhancing pathways between targeted brain regions. It is anticipated that this study will provide positive viewpoints for the neurorehabilitation of stroke patients based on the connection-level or circuit-level plasticity.

## Data Availability

The datasets generated during and/or analysed during the current study are available from the corresponding author on reasonable request.
